# Design and parameter optimization of a bilateral staggered wheel-spoon precision seed-metering device for millet plot breeding

**DOI:** 10.3389/fpls.2026.1967902

**Published:** 2026-10-27

**Authors:** Guoqiang Dun, Pengtao Lv, Yang He, Feng Jiang, Xinxin Ji, Ruiqi Wang, Ankang Mu

**Affiliations:** 1 Engineering Laboratory of Intelligent Agricultural Machinery Equipment, Harbin Cambridge University, Harbin, China; 2 College of Mechanical and Electrical Engineering, Northeast Forestry University, Harbin, China; 3 College of Control and Information Engineering, Northeast Forestry University, Harbin, China; 4 Inner Mongolia Chifeng Academy of Agriculture and Animal Husbandry Sciences, Chifeng, China

**Keywords:** agricultural machinery, millet, parameter optimization, plot breeding, seed-metering device

## Abstract

Precision seeding of micro-sized millet seeds in plot breeding faces critical challenges, including high miss-seeding rates and severe seed cross-contamination during variety changeovers. This study developed a novel bilateral staggered wheel-spoon precision seed-metering device to achieve precise hill-dropping of 5–8 seeds per hole, zero-residue rapid clearing, and high-precision low-speed seeding without seed damage. The device features a horizontal-axis bilateral staggered arrangement of 20 seed spoons, a lateral gravity seed-dropping mechanism and a semi-enclosed seed chamber. Discrete Element Method (DEM) simulation and Central Composite Design (CCD) response surface methodology were applied to optimize seed spoon cavity dimensions, followed by machine vision bench tests and field validation. Kinematic analysis verified that 20 staggered spoons effectively prevent centrifugal seed scattering. The optimized cavity has an effective depth of 4.4 mm, width of 4.1 mm and height of 2.7 mm, with a simulated seeding qualification rate above 92%. Bench tests at 9 rpm delivered a 92.67% qualification rate, 3.33% re-seeding rate and 4.00% miss-seeding rate. Field trials across three millet varieties maintained qualification rates ≥88%, with an 8.5-second zero-residue clearing process. This device resolves the conflict between micro-seed precision hill-dropping and efficient contamination-free clearing, fully satisfying the agronomic demands of millet plot precision breeding.

## 1 Introduction

Plot breeding experiments are a key link in the breeding of self-developed elite varieties and the safeguarding of food security (Shang et al., 2021). Different from large-field seeding, plot breeding trials require precision sowing of a fixed quantity of seeds of a single variety within a designated area, and impose strict requirements on seed mixing prevention, as well as core performance indicators including miss-seeding rate, re-seeding rate and hill spacing uniformity (Liu et al., 2011; St Jack et al., 2013). Foxtail millet (Setaria italica) is an important grain crop native to China, with abundant nutrients and mineral elements (Zhao and Diao, 2022; Liu et al., 2022). Compared with traditional drill seeding, precision hill seeding not only reduces seed consumption, but also facilitates nutrient uptake by seeds. Classified by working principles and technical characteristics, current global research on plot seed metering devices mainly focuses on three categories: pneumatic, mechanical, and novel electromechanical coupling seed metering technologies.

Pneumatic seed metering devices achieve gentle seed retention via airflow pressure differentials and exhibit low sensitivity to seed size. Researchers worldwide have realized high-precision metering and continuous automatic control of pneumatic seed metering systems under high-speed operation through PID closed-loop control (Shah et al., 2022), optimization algorithms (Karayel et al., 2022; Pareek et al., 2023), and the application of electronic control systems (Gong et al., 2011; Zhang et al., 2017). Novel centrifugal (Li et al., 2023), electromagnetic vibration (Zhang et al., 2024), and pneumatic–spoon interactive (Deng et al., 2024) seed metering devices explored in recent studies also show distinct advantages. However, such systems depend on high-precision airtight designs and complex piping structures. During frequent seed replacement in plot breeding, internal dead zones for seed retention are extremely prone to form, making machine cleaning highly difficult and thus failing to meet the residue-free and seed-mixing prevention requirements.

Mechanical seed metering devices are widely used in the breeding field due to their simple structure and low cost. Researchers have quantified the high-speed miss-seeding thresholds of fluted roller and cell wheel seed metering devices through simulation tests (Baghooee et al., 2023; Valentin et al., 2024). For rough-coated seeds or tiny grains (e.g., Chinese cabbage and foxtail millet), they optimized the spoon (cell) structure using active guide mechanisms (Ospanova et al., 2024) and the Discrete Element Method (DEM) (Zheng et al., 2024; Kou et al., 2024), thereby improving the seed filling qualified rate. Nevertheless, in order to forcibly adapt to tiny, irregular seeds, existing mechanical seed metering devices are often designed with overly complex cavities and restrictive seed protection belts, and these tiny clearances become dead zones for seed retention. Moreover, when rotational speed is increased to boost operation efficiency, foxtail millet seeds filled by passive gravity are highly susceptible to centrifugal force interference, causing seed splashing and miss-seeding in the seed protection zone.

Existing seed metering devices fail to achieve a balance between precision seeding of tiny foxtail millet grains and efficient machine cleaning for anti-seed-mixing in plot breeding. To address this issue, this study designs a bilateral staggered spoon-wheel precision seed metering device for foxtail millet plots, based on the physical properties of foxtail millet seeds and the agronomic requirements of plot breeding.

Compared with existing mechanical seed metering devices, the proposed device adopts a horizontal-shaft bilateral staggered arrangement, which reduces the upper limit of the seed metering disc rotational speed while maintaining seed dropping frequency, effectively mitigating centrifugal splashing interference for tiny grains. Combined with the side-opening seed dropping structure and semi-closed seed chamber design, it achieves gravity-driven gentle seed delivery during operation, as well as overall overturning for seed unloading and chamber cleaning during seed replacement. In recent years, several mechanical and pneumatic-assisted seed-metering devices have been developed specifically for small-grain crops, including spoon-wheel spinach seeders (Zheng et al., 2024), air-assisted millet seeders (Kou et al., 2024), electromagnetic vibration millet seeders (Zhang et al., 2024), and pneumatic-spoon interactive maize seeders (Deng et al., 2024). However, a horizontal comparison reveals three persistent gaps: (1) most reported devices retain single-sided seed spoon arrangements, which require high rotational speeds to achieve the target dropping frequency and thus induce centrifugal scattering of ultra-light millet seeds; (2) brush or scraper mechanisms are commonly used for seed cleaning, causing mechanical seed damage and leaving seed residues in narrow clearances that breed cross-contamination; and (3) internal dead zones for seed retention are difficult to access without full disassembly, making variety changeover slow and labor-intensive. The device proposed in this study addresses all three gaps simultaneously through a bilateral staggered horizontal-shaft layout, a side-opening gravity-driven seed-dropping mechanism without brushes, and a semi-enclosed chamber that can be completely flipped for residue-free unloading.

In this study, parameters of key components are determined via theoretical analysis. Simulations of the seed metering and seed unloading processes and response surface methodology (RSM) optimization are performed using EDEM discrete element software, followed by bench tests and field tests to validate the overall performance of the device. This study provides an engineering solution that integrates mechanical design, discrete-element simulation, and multi-objective optimization to solve the critical issues of high miss-seeding rates and seed mixing during machine cleaning in plot breeding of small-grain crops. The bilateral staggered wheel-spoon layout, the side-opening gravity-driven seed-dropping mechanism, and the flip-type residue-free unloading design represent the core engineering innovations of this work.

## 2 Overall structure and working principle of the seed metering device

### 2.1 Overall structure and seed unloading and chamber cleaning design

The overall structure of the proposed seed metering device is illustrated in **Figure 1**. Adopting a horizontal-shaft bilateral staggered seed metering configuration, the device is mainly composed of a fixed housing, a seed chamber housing, a main seed metering disc, and seed spoons mounted on both sides of the disc. The seed metering disc is positioned between the two housings. The fixed housing serves as the mounting interface for the seed metering device bracket and the stepper motor, while the two housings together form a semi-closed seed chamber.

**Figure 1:**
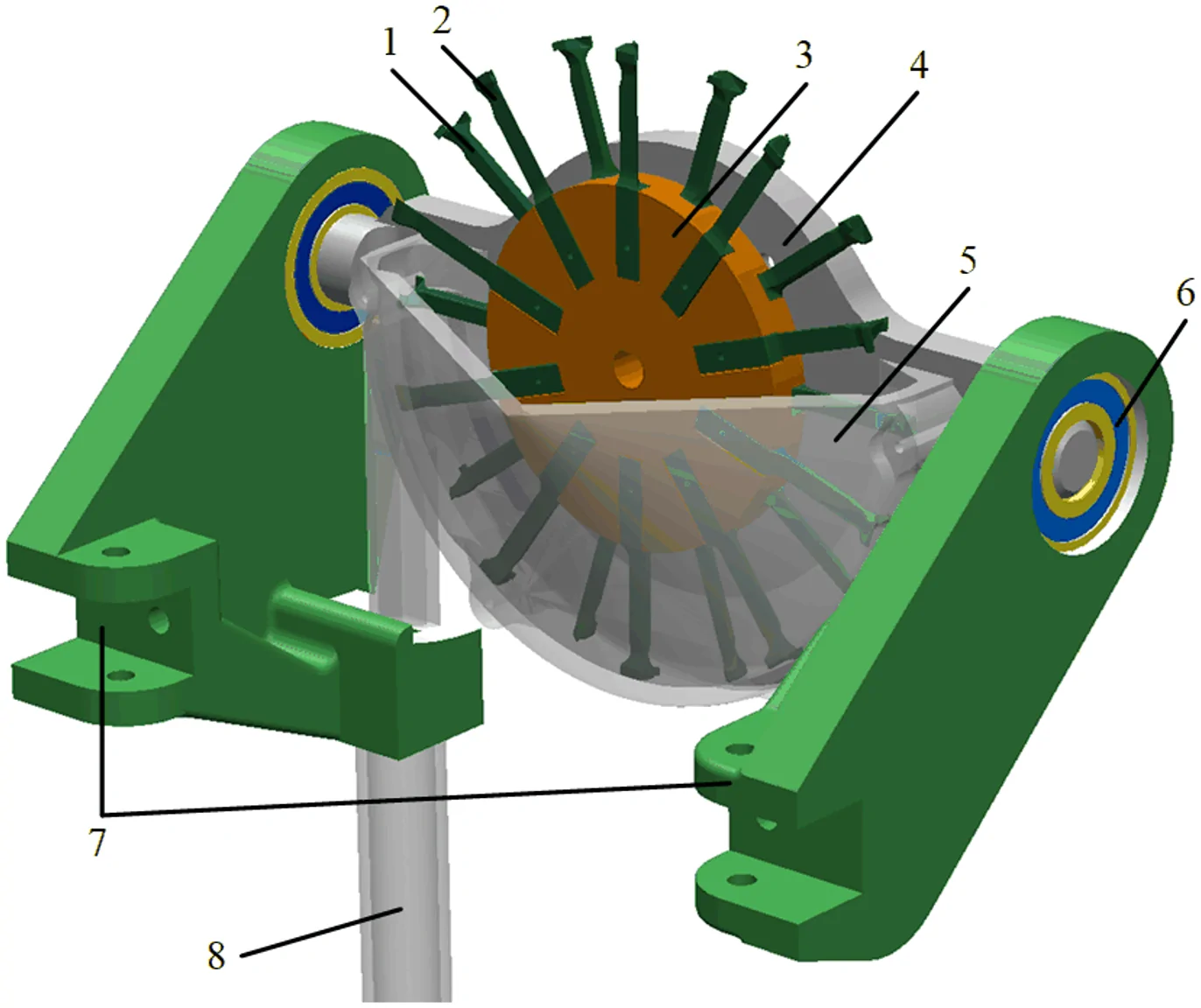
Overall structure of the seed-metering device (1. Inner seed spoon 2. Outer seed spoon 3. Main body of the seed disk 4. Fixed housing 5. Seed chamber housing 6. Bearing 7. Bracket of the seed-metering device 8. Seed guiding tube).

To eliminate seed mixing during seed replacement in plot breeding, the seed metering device adopts a dead-zone-free anti-residue structural design. The seed spoons remain tangent to the inner wall of the seed chamber housing at the mating surfaces, with the normal clearance precisely controlled, leaving no dead zones prone to seed retention inside the chamber. The fixed housing is rotatably connected to the seed metering device bracket via a bearing. For seed replacement in breeding experiments, rapid unloading of residual seeds and chamber cleaning can be achieved simply by rotating the entire seed metering device under gravity, without disassembly. A schematic diagram of the seed unloading and chamber cleaning mode of the proposed device is presented in **Figure 2**.

**Figure 2:**
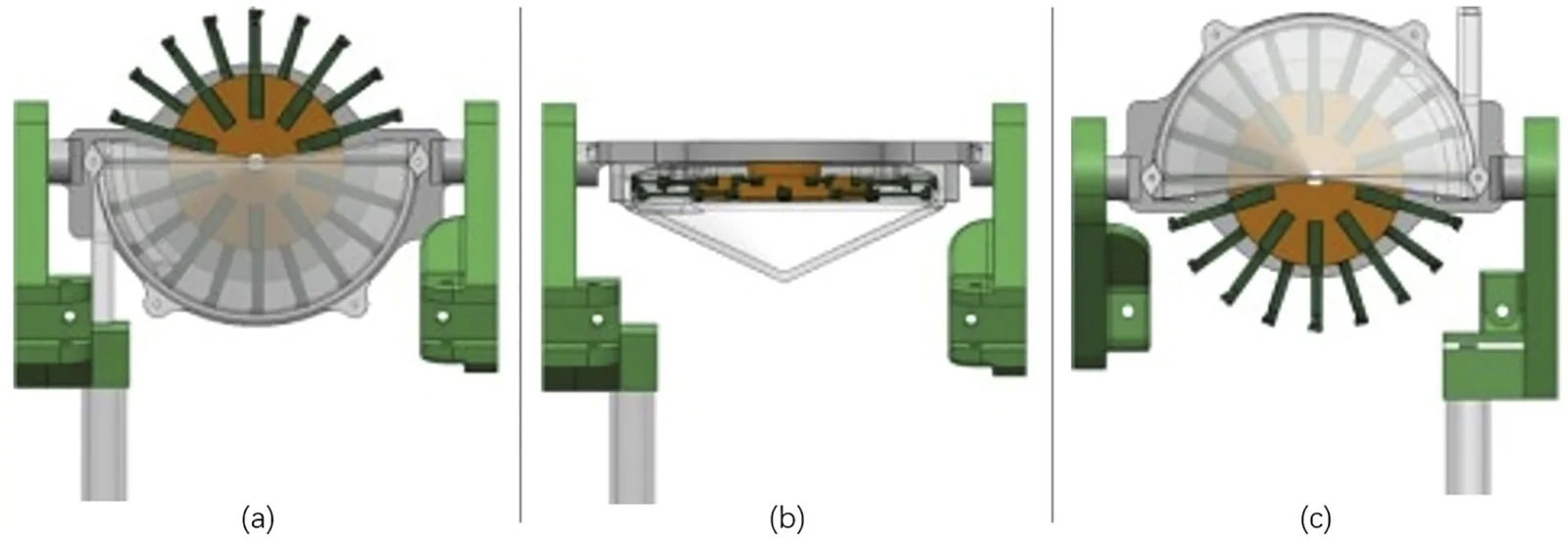
Schematic diagram of the clearing and unloading method of the seed-metering device: **(a)** Upright; **(b)** Flipping; **(c)** Inverted.

The seed metering device adopts a bilateral staggered arrangement of seed spoons, allowing it to meet the preset seeding frequency at a relatively low rotational speed and effectively avoid centrifugal force interference caused by high-speed operation. The seed spoons on both sides are symmetrically designed. Each seed spoon has an inward-sloping face, with a deflector arranged on its outer side wall. In the seed filling zone, the slope and deflector together drive the seed population to flow laterally; in the seed cleaning zone, excess seeds are removed by gravity along the slope. The inner wall of the seed chamber housing interfaces with the lateral opening of the seed spoon. In the non-seed-dropping zone, the open scoop body is enclosed to form a semi-closed seed holding cavity; in the seed dropping zone, a seed dropping window connected to the outside is provided on the seed chamber housing, which removes the lateral constraint on the seeds to complete the seed metering process. The profile structure of a single-side seed spoon is shown in **Figure 3** (the opposite side is a mirror image).

**Figure 3:**
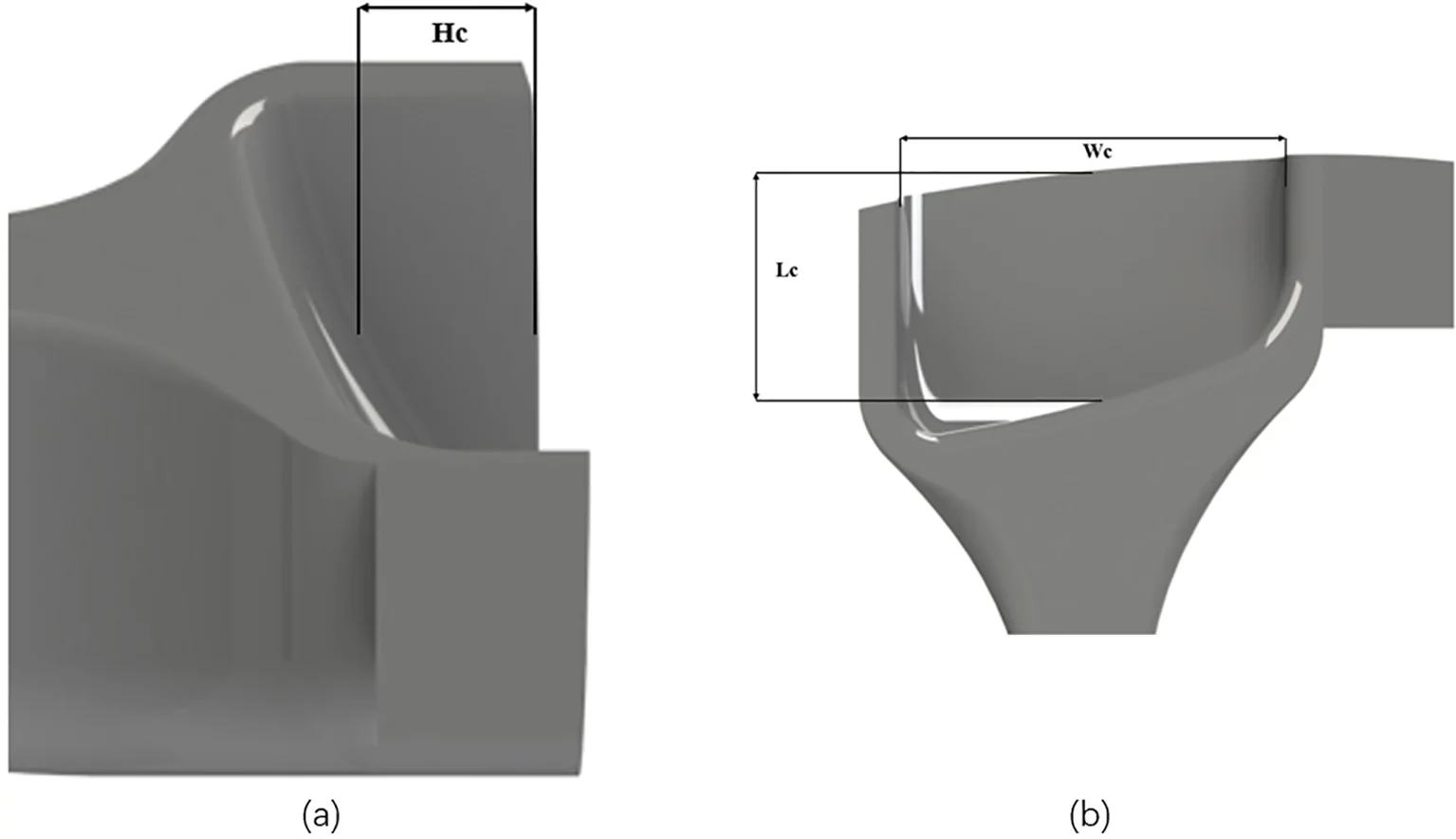
Outer structure diagram of the single-sided seed spoon: **(a)** Side view; **(b)** Top view.

### 2.2 Working principle of the seed metering device

During operation, seeds accumulate in the seed storage zone of the seed chamber. Driven by the motor, the seed metering disc rotates clockwise, carrying the seed spoons through four sequential working stages: seed filling, seed agitation and flow guidance, seed cleaning, and seed dropping. These four stages correspond to zones I–IV in **Figure 4**, respectively. Zone I (seed filling) is located at the bottom of the seed chamber where the seed population accumulates under gravity; Zone II (seed agitation and flow guidance) is the upward-curving section where the protruding deflector on each spoon redirects seeds laterally to prevent dead-zone accumulation; Zone III (seed cleaning) is the upper section where excess seeds slide back into the chamber under gravity along the cleaning edge; Zone IV (seed dropping) is the lateral opening window between 165° and 175° where seeds exit the cavity by lateral sliding. First, the seed spoons cut into the seed population at the bottom to complete seed filling. At the same time, their built-in inward-sloping faces and deflectors drive the surrounding seed population toward the opposite side, ensuring uniform and sufficient seed picking for all bilateral staggered seed spoons. Subsequently, as the seed spoons rotate upward into the seed cleaning zone, excess seeds resting on the edge of the spoon opening cannot remain balanced on the inward-sloping faces, and slide back down to the bottom of the seed chamber under the combined action of gravity and centrifugal force. Finally, when the seed spoons rotate to align with the seed dropping window, the lateral sealing constraint from the housing is removed. Without lateral support, the seeds inside the spoons slide out under gravity and centrifugal force and fall into the seed guide tube, completing one full seed metering cycle. The working principle of the proposed seed metering device is shown in **Figure 4**.

**Figure 4:**
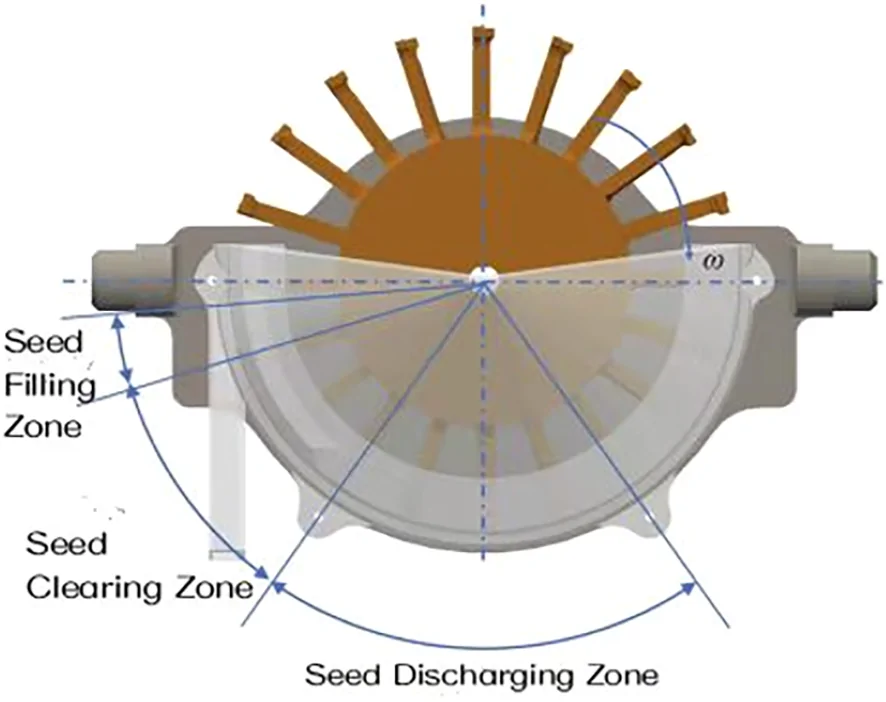
Schematic diagram of the working principle of the seed-metering device.

## 3 Design of key parameters and mechanical analysis of the seed metering device

### 3.1 Agronomic requirements and operating parameters

Northern China (including Northeast China, Inner Mongolia, and Hebei Province) is the primary producing area of foxtail millet in China. In this study, two Chinese local standards were referenced: Technical Regulation for Precision Hill Seeding of Zhangza Millet(DB1307/T 397–2023) and Technical Regulation for Mechanical Quantitative Hill Seeding Cultivation of Foxtail Millet(DB14/T 939–2024).According to the above standards, the agronomic parameters were determined as follows: the seeding method is precision hill seeding; the hill spacing is 20 cm (adjustable within 15–25 cm); the target seeding rate is 5–8 seeds per hill; and the seeding depth is 3–5 cm.

### 3.2 Dimension design of the seed metering disc

During operation of the seed metering device, each time a seed spoon completes a seed dropping cycle, the motor-driven seed metering disc delivers one hill of seeds into the furrow. To ensure accurate seed dropping frequency and precise operational synchronization, the number of seed spoons, the rotational speed of the seed metering disc, and the forward speed of the hand-pushed seeder shall meet the following kinematic relationship:

(1) $$n=\frac{60v_{m}}{ZS}$$

Note: n is the working rotational speed of the seed disk, r/min; *V_m_* is the manual forward speed, m/s; S is the hole spacing, m; Z is the total number of seed spoons.

Foxtail millet seeds have extremely low mass, and the requirement of filling 5–8 seeds per hill must be met. If the seed metering device is to achieve the preset high-frequency seeding requirement at a low rotational speed, increasing the number of seed spoons on a single side would lead to excessively narrow spacing between adjacent scoops, impairing the fluidity of the seed population in the seed filling zone. To address this, the seed spoons on both sides are arranged in a phase-staggered configuration, where each seed spoon on one side lies on the angular bisector of the central angle between two adjacent scoops on the opposite side. This structure doubles the actual seed dropping frequency per revolution of the seed metering disc compared with a single-side arrangement. Furthermore, the proposed horizontal-shaft bilateral staggered seed metering structure eliminates a common drawback of traditional mechanical seed metering devices, in which continuous impact from seed spoons during operation drives seeds to disperse into the non-seed-filling zone.

The main body of the seed metering disc is disc-shaped, with independent outward-extending seed spoons evenly distributed circumferentially along the edges of both sides. Let Z_1 be the number of evenly distributed seed spoons on one side of the disc; the total number of seed spoons is then Z = 2Z_1. To match the design requirements of overall dimensions (115 cm × 45 cm × 100 cm) and lightweight performance (15–25 kg), and to ensure a moderate disc volume with sufficient seed storage space in the inner cavity, the radius of the main seed metering disc body (excluding seed spoons) is preliminarily set at 40 mm. The seed spoons extend 30 mm outward from the disc via scoop arms, giving the seed metering disc a maximum rotary diameter of 140 mm.

### 3.3 Overall structural design of the seed spoon

The seed spoon is the core component that participates in seed filling, seed cleaning, and seed dropping. To improve seeding accuracy and ensure stable seed delivery quality of the metering device, this study designs the seed spoon as an asymmetric side-opening structure with smooth curved surface transitions. At the bottom of the seed spoon, there is a pocket-shaped blind hole. The inner wall of this region adopts smooth transitions between arcs and tangent lines (i.e., the seed dropping slideway), ensuring that 5–8 foxtail millet seeds remain stably clustered at the spoon bottom and are discharged at the corresponding angle. According to the grain size of foxtail millet, the seed dropping slideway is set to a fixed width of 2.2 mm. The back side wall above the seed spoon cavity features a distinct inward-downward inclined slope. As the seed spoon rotates upward with the disc, excess seeds that fail to enter the effective seed holding zone at the bottom slide smoothly down this slope under gravity, achieving gentle seed cleaning. A rectangular deflector, which protrudes prominently downward, is provided on the outer front edge of the seed spoon bottom on the side closer to the seed metering disc. At the moment the seed spoon cuts into the seed population, this protruding structure enhances seed filling fluidity by exerting lateral extrusion force on the seed mass, pushing excess seeds on the current side toward the opposite side. One side of the seed spoon is fully open and mates with the inner arc surface of the seed chamber housing. In the non-seed-dropping section, this open side maintains a micro-clearance fit with the smooth inner wall of the seed chamber housing, temporarily enclosing the side-opening scoop body into a semi-sealed cavity to prevent seed loss during the lifting process. In the seed dropping zone, the lateral constraint on seeds is released via the externally connected seed dropping window on the seed chamber housing, enabling gravity-driven seed dropping.

After the seed spoon cuts into the seed population in the bottom seed filling zone, excess seeds (beyond the target of 5–8 grains per hill) are often carried inside and outside the spoon due to internal friction and accumulation of the foxtail millet seed mass. To ensure the accuracy of precision hill seeding and improve the fluidity of the seed population, this study adopts a structural design that integrates a seed cleaning slope on the back side wall and a rectangular deflector on the outer front edge of the seed spoon. Traditional mechanical seed metering devices mostly use brushes or scrapers for forced seed cleaning, which can easily cause mechanical damage to foxtail millet seeds. In plot breeding scenarios, this structure also leads to mixing of different varieties, reducing experimental accuracy. To address these issues, a smooth inward-downward inclined slope (i.e., the seed cleaning slope) is designed on the back side wall above the seed spoon cavity. Leveraging the posture change of the seed metering disc during rotational lifting, this structure achieves non-destructive, gravity-driven seed cleaning. When the seed spoon rotates out of the seed population and lifts upward with the seed metering disc, the 5–8 seeds retained at the bottom of the seed spoon cavity are held stably under the geometric constraint of the cavity bottom. Meanwhile, excess seeds accumulated on the back side wall above the cavity and on top of the seeds inside the cavity slide back to the bottom of the seed chamber under gravity. To ensure that excess seeds under varying friction conditions slide off quickly and smoothly during the early lifting stage of the seed spoon, the inclination angle of the seed cleaning slope relative to the horizontal plane is set at 35°.

The foxtail millet seed population exhibits relatively poor fluidity under gravity and internal friction. To improve the seed filling quality of the metering device, a downward-protruding rectangular deflector is provided on the outer front edge of the seed spoon bottom adjacent to the seed metering disc. As the deflector sweeps through the seed mass, it exerts a lateral pushing force on the forward seed population, directing the seeds toward the opposite side. Combined with the aforementioned staggered arrangement of seed spoons on both sides, the seed population pushed by the deflector on one side enters the working trajectory of the next seed spoon on the opposite side that is about to enter the filling phase. This structure effectively eliminates single-side dead zones during continuous bilateral operation, improves the filling quality of seed spoons on both sides, and guarantees precision seed filling under low-speed operating conditions. An excessively large deflection angle would cause a sharp increase in frontal resistance when cutting into the seed population, easily leading to seed accumulation in front of the deflector and dispersion into the non-seed-filling zone. Conversely, an excessively small angle would generate insufficient lateral force, failing to push the seeds to the target position. Therefore, considering the flow guidance effect, the deflection angle between the rectangular deflector and the end face of the seed metering disc is set to 30°, with a deflector length of 2.7 mm.

### 3.4 Design of seed spoon arrangement and spoon cavity seed dropping slideway

The total number of seed spoons and their spatial arrangement on the seed metering disc not only affect the seed dropping frequency of the device, but also directly determine its operating rotational speed and angular velocity. An improper scoop count design would result in continuous impact of the spoons on foxtail millet seeds, causing the seed population to disperse from the seed filling zone to the non-seed-filling zone, or leading to miss-seeding in the seed dropping zone due to excessive angular velocity. In the seed filling and seed cleaning sections (during the upward rotation phase), the lateral open face of the seed spoon fits closely against the inner wall of the seed chamber housing, forming a semi-closed seed holding cavity that retains the seeds inside the spoon. When the seed spoon rotates into the seed dropping zone, the continuous lateral wall of the seed chamber housing ends at the seed dropping window. Without lateral support, the seeds slide along the seed dropping slideway of the spoon cavity into the window, and then enter the seed guide tube connected to the window to complete the seed dropping process. Therefore, the design of the seed dropping angle (i.e., the structural opening angle of the lateral seed dropping window) and the spoon cavity slideway jointly determine the trajectory and stability of seeds upon exiting the cavity.

Based on the kinematic relationship of the seeder established in the previous section, the angular velocity of the seed metering disc is related to the forward speed, hill spacing, and total number of seed spoons as follows as shown in Equation (2):

(2) $$\omega =\frac{2\pi v_{m}}{ZS}$$

Note: ω is the angular velocity of the seed disk, rad/s.

To determine the optimal number of seed spoons, this study conducts a force analysis of foxtail millet seeds under extreme seed dropping conditions. In the analysis, the 5–8 seeds inside the spoon cavity are simplified as an equivalent mass point of the seed cluster, with the rotation center of the seed metering disc taken as the coordinate origin. The proposed seed metering device adopts a gravity-driven seed dropping mechanism that releases lateral constraints on seeds via the lateral seed dropping window. In the seed dropping zone, the centrifugal force generated by the rotation of the seed metering disc is denoted as *F_c_*. The cluster of 5–8 foxtail millet seeds inside the seed spoon undergoes two simultaneous motions: tangential circular motion following the seed spoon, and outward lateral sliding along the seed dropping slideway of the spoon cavity to complete the seed dropping process.

If the rotational speed of the seed metering disc is excessively high, the seed spoon will sweep past the seed dropping window too quickly, resulting in miss-seeding. Accordingly, the kinematic condition for successful seed dropping is defined as follows: the time required for seeds to slide laterally out of the spoon cavity must be shorter than the time available for the seed spoon to pass through the seed dropping window. The available sliding time during the full passage of the seed spoon through the window is given by Equation (3):

(3) $$t_{\omega }=\frac{\alpha_{\omega }}{\omega }$$

Note: tω is the time provided for the seeds to slide off as the seed spoon completely passes through the window, s; αω is the seed-dropping angle, rad.

To facilitate subsequent mechanical analysis, appropriate simplifications are applied to the force model. The seed cluster is assumed to be located on the same horizontal line as the coordinate origin (i.e., the rotation center of the seed metering disc). This simplification not only reduces computational complexity, but also imposes a more stringent constraint on performance validation, yielding conservative and reliable results.

At the position where the cavity bottom is at the same horizontal level as the rotation center (i.e., the horizontal position of the seed-dropping zone), gravity acts vertically downward, while the centrifugal force Fc generated by the rotation of the seed metering disc acts horizontally outward. The normal pressure on the seed cluster on the seed dropping slideway equals the difference between the gravity component and the centrifugal force component. Only the case where the gravity component exceeds the centrifugal force component is considered in this study. Accordingly, the resulting sliding friction resistance is given by Equation (4):

(4) $$f=\mu_{s}N_{n}=\mu_{s}(F_{n}-F_{c})=\mu_{s}(m_{t}g\cos\gamma -m_{t}\omega^{2}R_{e}\sin\gamma )$$

Note: *µ_s_* is the static friction coefficient between the millet seeds and PLA; *Nn* is the normal support force exerted on the seed group on the cavity slide, N; *Fn* is the normal component of gravity (pointing towards the bottom of the spoon), N; *m_t_* is the total mass of the point mass representing the 5~8 millet seeds group, kg; *γ* is the included angle between the seed spoon cavity slide and the horizontal plane,(°); *F_c_* is the component of the centrifugal force generated by the rotation of the seed disk, N; *R_e_* is the distance from the bottom of the cavity to the center of rotation, m.

By Newton’s second law, the resultant force that drives the lateral sliding of the seed cluster is given by (the acceleration component of the centrifugal force along the seed dropping slideway is neglected to ensure computational reliability):

(5) $$m_{t}a_{s}=m_{t}g\sin\gamma -\mu_{s}(m_{t}g\cos\gamma -m_{t}\omega^{2}R_{e}\sin\gamma )$$

Physically, (Equation 5) states that the net driving force for lateral seed sliding equals the tangential component of gravity along the slideway minus the sliding friction force. The centrifugal force contributes only through its normal component, which presses the seed cluster against the cavity surface and thereby increases the friction force that opposes sliding. This formulation captures the essential trade-off: gravity promotes seed release, while rotation-induced normal pressure suppresses it. After simplification, the sliding acceleration (*a_s_*) is given by:

(6) $$a_{s}=g(\sin\gamma -\mu_{s}\cos\gamma )+\mu_{s}\omega^{2}R_{e}\sin\gamma$$

(Equation 6) reveals that the sliding acceleration is positive only when the tangential gravity component exceeds the friction force. Since the friction coefficient *μ_s_* and the slideway angle γ are fixed for a given design, the acceleration decreases as rotational speed ω increases because the centrifugal normal term *μ_s_* mt Re ω² grows. This establishes a direct physical link between rotational speed and seed-dropping reliability: higher speed reduces the driving acceleration available for seed release. Accordingly, the lateral sliding distance *L_a_* required for the seed cluster to exit the spoon cavity is given by Equation (7):

(7) $$L_{a}=\frac{1}{2}a_{s}t_{s}^{2}$$

Note: *t_s_* is the lateral sliding time, s.

For successful seed dropping, the time required for the seed cluster to slide laterally out of the spoon cavity must be no greater than the available sliding time for seeds during the full passage of the seed spoon through the seed dropping window, that is:

(8) $$2L_{a}\omega^{2}\leq \alpha_{\omega }^{2}[g(\sin\gamma -\mu_{s}\cos\gamma )+\mu_{s}\omega^{2}R_{e}\sin\gamma ]$$

(Equation 8) is the core seed-dropping constraint: the time required for the seed cluster to slide across the cavity exit distance La must not exceed the time window during which the seed spoon is aligned with the lateral dropping opening. If *t_s_* > *t_ω_*, the seed spoon rotates past the dropping window before the seeds have exited, resulting in seeds being carried back into the seed chamber and a subsequent miss-seeding event. This constraint directly determines the maximum allowable rotational speed for reliable metering. The upper limit of the critical angular velocity is given by:

(9) $$\omega \leq \alpha_{\omega }\sqrt{\frac{g(\sin\gamma -\mu_{s}\cos\gamma )}{2L_{a}-\alpha_{\omega }^{2}\mu_{s}R_{e}\sin\gamma }}$$

(Equation 9) defines the seed-dropping critical angular velocity ω_max_. Below this value, the sliding time *t_s_* is shorter than the available window time *t_ω_*, ensuring reliable seed release. Above ω_max_, the available window time becomes too short for complete seed evacuation, and the miss-seeding rate increases sharply. This threshold therefore sets the upper operating boundary for the metering device. The lower limit of the total number of seed spoons is given by:

(10) $$Z\geq \frac{2\pi v_{m}}{S\alpha_{\omega }}\sqrt{\frac{2L_{a}-\alpha_{\omega }^{2}\mu_{s}R_{e}\sin\gamma }{g(\sin\lambda -\mu_{s}\cos\gamma )}}$$

(Equation 10) gives the minimum total number of seed spoons required to maintain the target hill-dropping frequency at a rotational speed below ω_max_. A larger number of spoons allows the same dropping frequency to be achieved at a lower rotational speed, which increases the available window time *t_ω_* and improves filling reliability. Conversely, too few spoons force a higher rotational speed to maintain frequency, pushing the device closer to or beyond the critical speed and increasing miss-seeding. This equation therefore links the structural parameter (spoon count) directly to the operational speed constraint. Based on the maximum operating conditions of the seeder (maximum forward speed *V_m_* = 0.8m/s, hill spacing *S* = 0.2m, and working radius *R_e_* = 0.07”m”, which is derived from the maximum radius of the seed metering disc), and the sliding friction coefficient between foxtail millet seeds and PLA (Polylactic Acid) material *μ_s_* = 0.6224, the following parameters are preliminarily determined. Based on the outer diameter of the seed metering disc (0.14 m) and the lateral sliding distance of the seed cluster *L_a_* = 0.015m, the seed dropping angle *α_ω_* is set at 10°, and the inclination angle between the seed dropping slideway of the spoon cavity and the horizontal plane is set at *γ* = 50°.

Substituting the aforementioned extreme operating condition parameters into the seed dropping constraint (Equation 10) yielded the following results: to prevent miss-seeding caused by seed spoons sweeping past the seed dropping window, the seed-dropping critical rotational speed is 18.5 r/min, and the total number of seed spoons must exceed 12.94.

To ensure a sufficient safety margin for the seed metering device under complex field conditions (e.g., machine vibration or instantaneous fluctuations in forward speed), this study sets the total number of seed spoons at 20. The spoons are arranged in a bilateral phase-staggered configuration, with 10 scoops on each side and a phase offset of 18° between the two sides. This design value exceeds the theoretical lower limit to prevent miss-seeding, avoids miss-seeding caused by excessive centrifugal force and rotational speed, and effectively guarantees the qualified rate of clustered hill seeding for foxtail millet.

This study considers both the force stability of foxtail millet seed clusters within the spoon cavity and the seed dropping speed. Based on the aforementioned calculation results for the 10° seed dropping angle, the lateral seed dropping window is positioned within the range of 165° to 175° in the clockwise rotational coordinate system. At this phase, the centrifugal force acting on the seed cluster in the cavity is directed outward from the seed metering disc, while the component of gravity along the lateral seed dropping slideway is relatively high, thereby reducing the seed dropping duration.

It is verified that with 20 seed spoons, even under the maximum operating condition at a forward speed of 0.8 m/s, the operating rotational speed of the seed metering disc is only 12 r/min. Under this condition, the normal component of the centrifugal force accounts for only 1.34% of the normal component of gravity, which is far below the critical threshold (corresponding to a normal-force critical rotational speed of approximately 103.5 r/min). Therefore, within the parameter range of this study, the normal force remains consistently positive and sliding friction is always present, making it unnecessary to discuss the failure mode of a non-positive normal force.

The above analysis adopts a conservative simplification by treating the 5–8 seed cluster as a point mass located at the same horizontal level as the rotation center. In reality, the seed cluster rests at the cavity bottom, which deviates slightly from the rotation center in the radial direction. To assess the error boundary introduced by this simplification, a sensitivity analysis was performed by varying the effective rotation radius Re from 0.06 m to 0.08 m, bracketing the actual cavity-bottom position within the 0.07 m design radius. The calculated seed-dropping critical rotational speed remains within a narrow range of 18.51–18.60 r/min, and the theoretical minimum spoon count varies only slightly from 12.90 to 12.97. The selected design value of 20 spoons thus provides a safety margin of approximately 54% above the lower bound across the entire range. Separately, the normal-force critical speed (above which the normal support force becomes non-positive) varies from 96.9 to 111.9 r/min over the same Re range, but remains far above the working speed of 12 r/min. These results confirm that the conservative point-mass assumption does not materially alter the design conclusion and provides an adequate safety margin for field vibration and forward-speed fluctuations.

### 3.5 Theoretical dimensional design of the seed spoon cavity

The seed spoon is the key component of the seed metering device for seed filling and dropping, and the structural parameters of the spoon cavity are critical determinants of seed filling, cleaning, and metering quality. To facilitate subsequent design and optimization, the structural parameters of the spoon cavity are defined as the effective cavity depth Lc, effective cavity width *_Wc_*, and effective cavity height *H_c_*. The effective cavity depth is defined as the distance from the center of the spoon cavity bottom to the highest point on the inner side of the seed spoon, which directly determines the number of seed layers that can be stably held within the cavity. The effective cavity width is defined as the distance between the inner surfaces of the two side walls of the seed spoon. The effective cavity height is defined as the distance from the center of the open lateral end face of the seed spoon to the center of the back closed wall.

Based on experimental observations and previous studies (Yang et al., 2025), foxtail millet seeds are generally small and have a semi-ellipsoidal shape, with an arched dorsal side and a relatively flat ventral side. Their filling postures inside the seed metering device are distinct from those of spherical seeds. For ellipsoidal seeds, three typical filling postures exist in the seed metering device, namely tangential, radial, and axial orientations (Zhang, 2014; Du et al., 2019).

Let the average volume of a single foxtail millet seed be V_0_. Based on the empirical geometric formula, it is given by Equation (11):

(11) $$V_{0}=\frac{\pi }{6}L_{ave}W_{ave}T_{ave}$$

Note: *V_0_* is the average volume of a single millet seed, mm³; *L_ave_* is the average length of the millet seeds, mm; *W_ave_* is the average width of the millet seeds, mm; *T_ave_* is the average thickness of the millet seeds, mm.

According to measurement data from previous studies, the mean dimensions of a single foxtail millet seed are 2.061 mm in length, 1.753 mm in width, and 1.342 mm in thickness, yielding an average volume of approximately 2.53 mm³ per seed. To achieve stable filling of 5 to 8 seeds, the total volume of the seed cluster ranges from 12.65 mm³ to 20.24 mm³. Since seeds are not densely packed when filling into the spoon during low-speed rotation, natural stacking voids exist between individual seeds. As reported in the literature (Balasubramanian and Viswanathan, 2010; Singh et al., 2010), the packing porosity of small-grain millet seeds with a moisture content of approximately 15% ranges from 0.45 to 0.60. Although Singh et al. (2010) specifically studied barnyard millet (Echinochloa crus-galli), foxtail millet (Setaria italica) and barnyard millet share similar semi-ellipsoidal seed geometry and comparable bulk density, so their packing porosities are expected to fall within a comparable range. To ensure that the spoon cavity can smoothly accommodate the entire target seed cluster, the theoretically designed effective volume of the spoon cavity shall satisfy Equation (12):

(12) $$V_{c}=\frac{V_{5-8}}{1-\epsilon }$$

Note: *V_c_* is the theoretical designed effective capacity of the seed spoon cavity, mm³; *V_5–8_*is the net total volume of 5~8 seeds (excluding pores), mm³; *ϵ* is the porosity of the seed pile.

The effective cavity volume required for clustered filling of 5–8 foxtail millet seeds is calculated to be in the range of 23.00–50.60 mm³. Based on the volume requirement and the seed arrangement pattern at the spoon bottom, the cavity dimensions are designed to ensure smooth entry of the seed cluster during filling and to prevent seed jamming during lateral dropping. Specifically, the effective cavity width shall accommodate 2 to 2.5 seeds, while both the effective depth and effective height shall accommodate 1.5 to 2.5 seeds each. The corresponding geometric constraints shall satisfy Equation (13):

(13) $$\left\{ \begin{array}{c} 1.5L_{\max}<L_{c}<2.5L_{\max}\\ 2W_{\max}<W_{c}<2.5W_{\max}\\ 1.5T_{\max}<H_{c}<2.5T_{\max} \end{array}\right.$$

Note: *L_c_* is the effective depth of the cavity, mm; *W_c_* is the effective width of the cavity, mm; *H_c_* is the effective height of the cavity, mm; *L_max_* is the maximum length of the millet seeds, mm; *W_max_* is the maximum width of the millet seeds, mm; *T_max_* is the maximum thickness of the millet seeds, mm.

Among the structural parameters, the dimensions of the seed spoon cavity are highly sensitive factors that directly determine seeds per hill. An oversized cavity may hold more than 8 seeds during filling, resulting in re-seeding and subsequent labor-intensive thinning. Conversely, an undersized cavity will have a seed filling quantity of fewer than 5 seeds, which easily leads to seedling gaps in field production. Based on preliminary calculations and geometric constraints, the initial design ranges of the spoon cavity parameters are determined as follows: effective cavity depth of 3.3–5.5 mm, effective cavity width of 3.5–4.5 mm, and effective cavity height of 2.0–3.5 mm. To ensure the validity of the optimal dimensions and high seeding accuracy, this study will extract the key structural parameters and further determine the optimal three-dimensional combination of the seed spoon through EDEM 2023 discrete element simulations and response surface optimization tests in the following sections.

## 4 Seed metering performance analysis and parameter optimization based on discrete element method

### 4.1 Establishment of simulation model and parameter settings

The seed metering devices used in subsequent bench and field tests are fabricated by 3D printing. Accordingly, PLA is set as the material of all components in contact with foxtail millet seeds during the simulation. Since no cohesion occurs between individual seeds or between seeds and PLA material, the Hertz–Mindlin (no-slip) contact model is selected as the simulation contact model for both seed–seed interactions and seed–device interactions, based on previous studies (Dun et al., 2019; Zhao et al., 2017). Based on related studies, the discrete element model of foxtail millet seeds, the physical parameters of the seeds and seed metering device materials, and the corresponding contact mechanical parameters are determined. The detailed simulation parameters are listed in **Table 1**.

**Table 1:** Discrete element simulation parameters of the seed-metering device.

Type	Parameter	Value
Millet seeds	Poisson’s ratio	0.39
	Shear modulus/Pa	5.252×105
	Density/kg·m-3	1054
Seed-metering device	Poisson’s ratio	0.30
	Shear modulus/Pa	1.04×107
	Density/kg·m-3	1290
Millet seeds-Millet seeds	Coefficient of restitution	0.512
	Static friction coefficient	0.22
	Rolling friction coefficient	0.041
Millet seeds-Seed-metering device	Coefficient of restitution	0.475
	Static friction coefficient	0.6224
	Rolling friction coefficient	0.1

The seed–PLA contact parameters (static friction coefficient SFC = 0.6224, rolling friction coefficient RFC = 0.10, coefficient of restitution = 0.475) were directly adopted from the DEM calibration of foxtail millet seeds conducted by Yang et al. (2025), which used the same PLA material and comparable seed moisture content (≈15%). The validity of this parameter set is supported by the close agreement between simulation and bench test results (Section 4), where the simulated qualification rate differs from the bench measurement by less than 1 percentage point.

The dimensions of the seed spoon cavity are set to the mid-values of the structural parameter ranges determined via theoretical analysis. A three-dimensional model of the seed metering device is constructed using SolidWorks 2022. To improve computational efficiency, the model is simplified without compromising simulation accuracy and then imported into EDEM 2023 in STL format. The material properties and inter-material contact parameters are configured according to the simulation parameters listed in **Table 1**. A dynamic particle factory is arranged above the seed chamber housing. The foxtail millet particle model and corresponding settings adopt the calibrated model and parameters described in the previous section, with a total of 800 seeds generated at a rate of 5000 seeds per second. A constant rotational motion is applied to the seed metering disc, starting at 0.5 s with a rotational speed of 12 r/min. The simulation fixed time step is set to 4.15338 × 10⁻^7^ s (approximately 15% of the Rayleigh time step), and the target data saving interval is set to 0.01 s. For each simulation run, seed-dropping events were continuously recorded over a sufficient period after the disc reached stable rotation, ensuring that at least 150 hills were counted per run to guarantee statistical reliability. The simulation model of the seed metering device is shown in **Figure 5**. In **Figure 5**, label 1 indicates the foxtail millet seed particles generated by the dynamic particle factory, label 2 indicates the seed metering disc with bilateral staggered seed spoons, and the surrounding purple structure (label 3) is the seed-metering device housing that encloses the filling, cleaning, and dropping zones.

**Figure 5:**
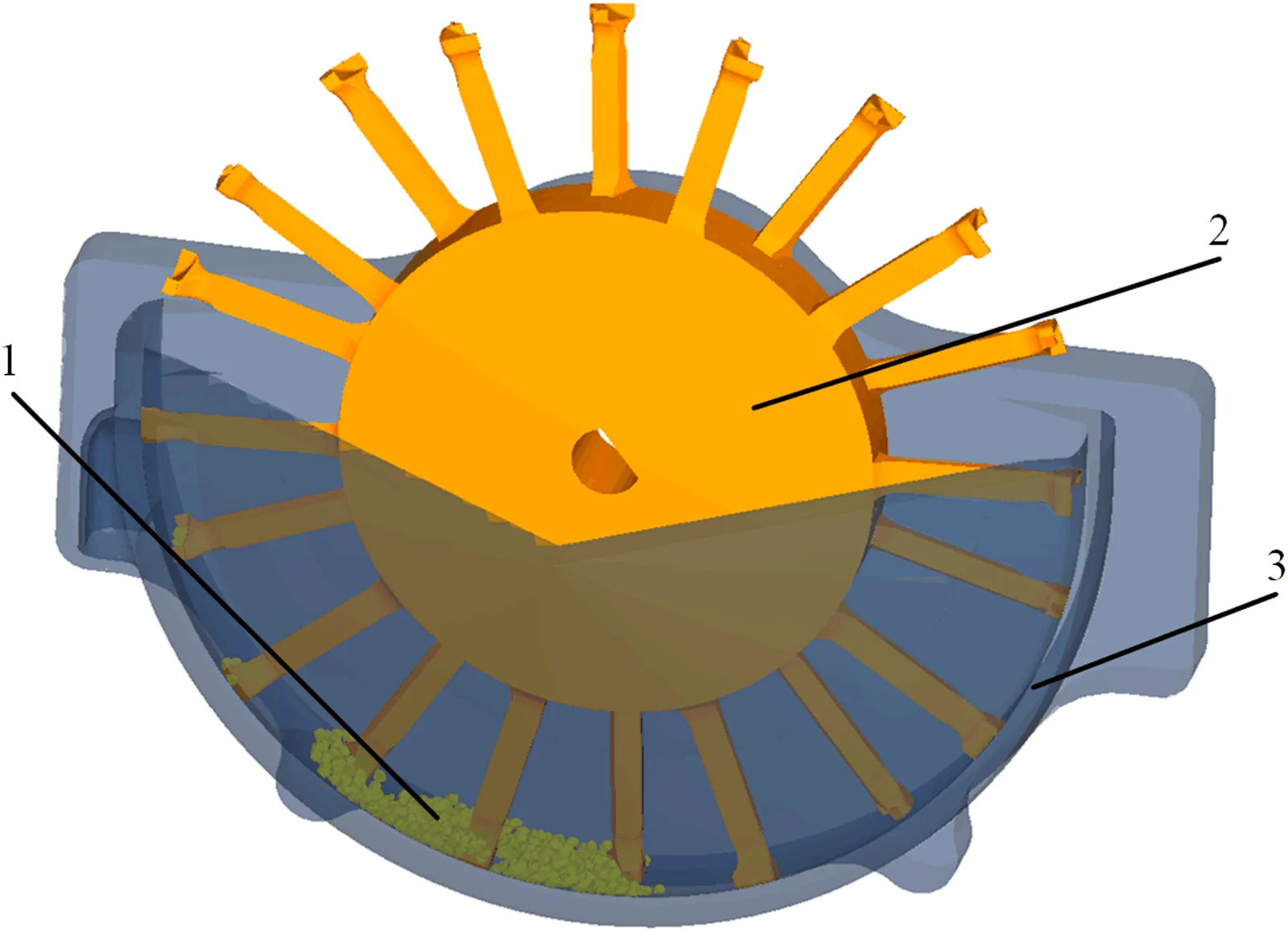
Simulation model of the seed-metering device (1. Millet seeds 2. Seed disk 3. Seed-metering device housing).

### 4.2 Experimental factors and evaluation indicators for simulation optimization tests

The effective cavity depth, effective cavity width, and effective cavity height are selected as the key experimental factors. Based on the aforementioned theoretical calculations, the preliminary design ranges are set as 3.3–5.5 mm for effective cavity depth, 3.5–4.5 mm for effective cavity width, and 2.0–3.5 mm for effective cavity height.

In accordance with the Test Methods for Single-seed (Precision) Seeders(GB/T 6973-2005), the Technical Specifications for Single-seed (Precision) Seeders (JB/T 10293-2013), and previous studies, combined with the previously determined agronomic requirements, the performance evaluation indicators are defined as follows: the qualified rate of seeds per hill (5–8 seeds per hill is defined as qualified), the re-seeding rate (more than 8 seeds per hill is defined as re-seeding), and the miss-seeding rate (fewer than 5 seeds per hill is defined as miss-seeding). The corresponding formulas are given by:

(14) $$\left\{ \begin{array}{c} Q=\frac{n_{q}}{N}\times 100\%\\ R=\frac{n_{r}}{N}\times 100\%\\ M=\frac{n_{m}}{N}\times 100\% \end{array}\right.$$

Note: *N* is the total number of seeded holes; *n_q_* is the number of qualified holes; *n_r_* is the number of re-seeded holes; *n_m_* is the number of miss-seeded holes.

### 4.3 Single-factor tests

Given the structural characteristics of the seed metering device, the design of the spoon cavity directly affects its operating performance. To determine the optimal dimensional parameters of the spoon cavity, single-factor simulation tests are designed to investigate the effects of effective cavity depth, effective cavity width, and effective cavity height on the qualified rate of seeds per hill, the re-seeding rate, and the miss-seeding rate, respectively. The single-factor test results are presented in **Table 2**.

**Table 2:** Statistical results of the single-factor tests.

Test factor	Parameter level/mm	Qualification rate/%	Re-seeding rate/%	Miss-seeding rate/%
Effective depth of the cavity	3.30	75.58	2.75	21.67
	3.85	81.08	5.58	13.33
	4.40	85.83	10.25	3.92
	4.95	81.92	16.42	1.67
	5.50	76.67	22.50	0.83
Effective width of the cavity	3.50	76.08	3.92	20.00
	3.75	80.25	6.42	13.33
	4.00	83.33	8.33	8.33
	4.25	85.25	10.58	4.17
	4.50	78.08	20.25	1.67
Effective height of the cavity	2.05	76.92	3.92	19.17
	2.40	81.67	6.67	11.67
	2.75	85.58	10.25	4.17
	3.10	82.75	14.75	2.50
	3.45	77.5	20.83	1.67

As shown in **Table 2**, across all three structural parameters of the spoon cavity, the qualified rate of seeds per hill consistently follows a trend of first increasing and then decreasing as the parameter level rises.

For the effective cavity depth, the qualified rate reaches its optimal value of 85.83% at 4.40 mm. Considering interactions between factors in subsequent optimization, the reasonable range of effective cavity depth is determined as 3.85–4.95 mm. For the effective cavity width, the maximum qualified rate of 85.25% occurs at 4.25 mm, and its reasonable range is accordingly determined as 3.75–4.25 mm. For the effective cavity height, the qualified rate peaks at 85.58% when the parameter is set to 2.75 mm, with the corresponding reasonable range defined as 2.40–3.10 mm.

### 4.4 Response surface optimization tests based on central composite design

To investigate the effects of effective cavity depth, width, height, and their interactions on the operating performance of the seed metering device and to identify the optimal structural parameter combination, multi-factor simulation optimization is conducted using central composite design (CCD). Consistent with the single-factor tests, effective cavity depth, width, and height are selected as the key experimental factors, with performance evaluation indicators identical to those of the single-factor tests. The factor-level coding and experimental levels are presented in **Table 3**.

**Table 3:** Test factors and level codes.

Code	Test factor		
	A: Effective depth of the cavity/mm	B: Effective width of the cavity/mm	C: Effective height of the cavity/mm
-1.682(-α)	3.85	3.75	2.4
-1	4.07	3.85	2.54
0	4.40	4.00	2.75
1	4.73	4.15	2.96
1.682(+α)	4.95	4.25	3.10

The experimental scheme is generated using Design-Expert 13. The test arrangements and corresponding simulation results are presented in **Table 4**.

**Table 4:** Test scheme and results.

Code	Test factor			Qualification rate/%	Re-seeding rate/%	Miss-seeding rate/%
	A/mm	B/mm	C/mm			
1	4.07	3.85	2.54	83.76	1.04	15.2
2	4.73	3.85	2.54	85.55	5.53	8.92
3	4.07	4.15	2.54	88.96	1.47	9.57
4	4.73	4.15	2.54	89.54	8.90	1.56
5	4.07	3.85	2.96	83.85	1.67	14.48
6	4.73	3.85	2.96	85.11	9.09	5.80
7	4.07	4.15	2.96	88.85	3.42	7.73
8	4.73	4.15	2.96	86.51	13.15	0.34
9	3.85	4	2.75	85.38	0.88	13.74
10	4.95	4	2.75	86.15	12.51	1.34
11	4.4	3.75	2.75	84.95	1.84	13.21
12	4.4	4.25	2.75	91.92	7.06	1.02
13	4.4	4	2.4	86.89	1.83	11.28
14	4.4	4	3.1	86.76	7.62	5.62
15	4.4	4	2.75	90.78	4.61	4.61
16	4.4	4	2.75	91.95	3.56	4.49
17	4.4	4	2.75	92.35	4.30	3.35
18	4.4	4	2.75	91.86	4.72	3.42
19	4.4	4	2.75	92.04	4.29	3.67
20	4.4	4	2.75	92.03	4.62	3.35

Analysis of variance (ANOVA) is conducted on the fitted quadratic regression model, and the results are presented in **Table 5**.

**Table 5:** Analysis of variance for the regression models of test indices.

Source	Freedom	Qualification rate/%		Re-seeding rate/%		Miss-seeding rate/%	
		F	P	F	P	F	P
Model	9	68.52	<0.0001	125.89	<0.0001	82.7	<0.0001
A	1	1.69	0.2233	797.7	<0.0001	328.25	<0.0001
B	1	188.18	<0.0001	114.07	<0.0001	261.38	<0.0001
C	1	3.47	0.0921	136.66	<0.0001	33.74	0.0002
AB	1	9.96	0.0102	15.87	0.0026	0.0414	0.8429
AC	1	5.13	0.047	15.75	0.0026	0.6769	0.4298
BC	1	3.35	0.097	2.33	0.1582	0.13	0.726
A2	1	237.72	<0.0001	50.08	<0.0001	42.89	<0.0001
B2	1	76.85	<0.0001	0.3711	0.556	33.68	0.0002
C2	1	163.26	<0.0001	1.96	0.1914	66.36	<0.0001
Residual	10						
Lack of Fit	5	0.9716	0.5122	1.39	0.3628	2.45	0.1742
Pure Error	5						
Total	19						

The ANOVA results show that the effective cavity depth (A), width (B), and height (C) significantly affect both the re-seeding rate and miss-seeding rate, with p values as low as 0.0002. The qualified rate is primarily influenced by the effective cavity width (B). The interaction between A and B exhibits a significant nonlinear effect on both the qualified rate and the re-seeding rate. The quadratic terms A^2, B^2, and C^2 are all highly significant for the miss-seeding rate. These results indicate that the selected parameter ranges are reasonable and that the response model contains obvious optimal regions.

Nonsignificant terms in the regression equations are eliminated. Meanwhile, the main effect terms that serve as lower order terms for significant interaction terms and quadratic terms are retained to ensure fitting accuracy and maintain the hierarchy of the regression model. The resulting quadratic polynomial regression equations, expressed in coded values for the qualified rate of seeds per hill, re-seeding rate, and miss-seeding rate as functions of effective cavity depth (A), width (B), and height (C), are given as follows in Equations (15–17):

(15) $$Q=91.84+0.1893A+2B-0.2716C-0.6012AB-0.4312AC-2.19A^{2}-1.24B^{2}-1.81C^{2}$$

(16) $$R=4.53+3.56A+1.35B+1.47C+0.6562AB+0.6537AC+0.8462A^{2}$$

(17) $$M=3.81-3.75A-3.35B-1.2C+1.32A^{2}+1.17B^{2}+1.64C^{2}$$

**Figure 6** presents the response surface plots illustrating the effects of different factors on the qualified rate of seeds per hill and the re-seeding rate. The results show that the interactions between cavity parameters have significant effects on seed metering performance. For the qualified rate, the depth–width and depth–height interactions both exhibit a clear trend of first increasing and then decreasing. The maximum qualified rate occurs in the central region where the depth ranges from 4.30 mm to 4.50 mm and the height varies from 2.70 mm to 2.80 mm. As for the re-seeding rate, it rises sharply with increasing values of each dimensional parameter. Among these factors, the re-seeding rate is most sensitive to variations in cavity depth. Therefore, maintaining the cavity parameters at a moderate to low level is essential to simultaneously achieve a high qualified rate and a low re-seeding rate.

**Figure 6:**
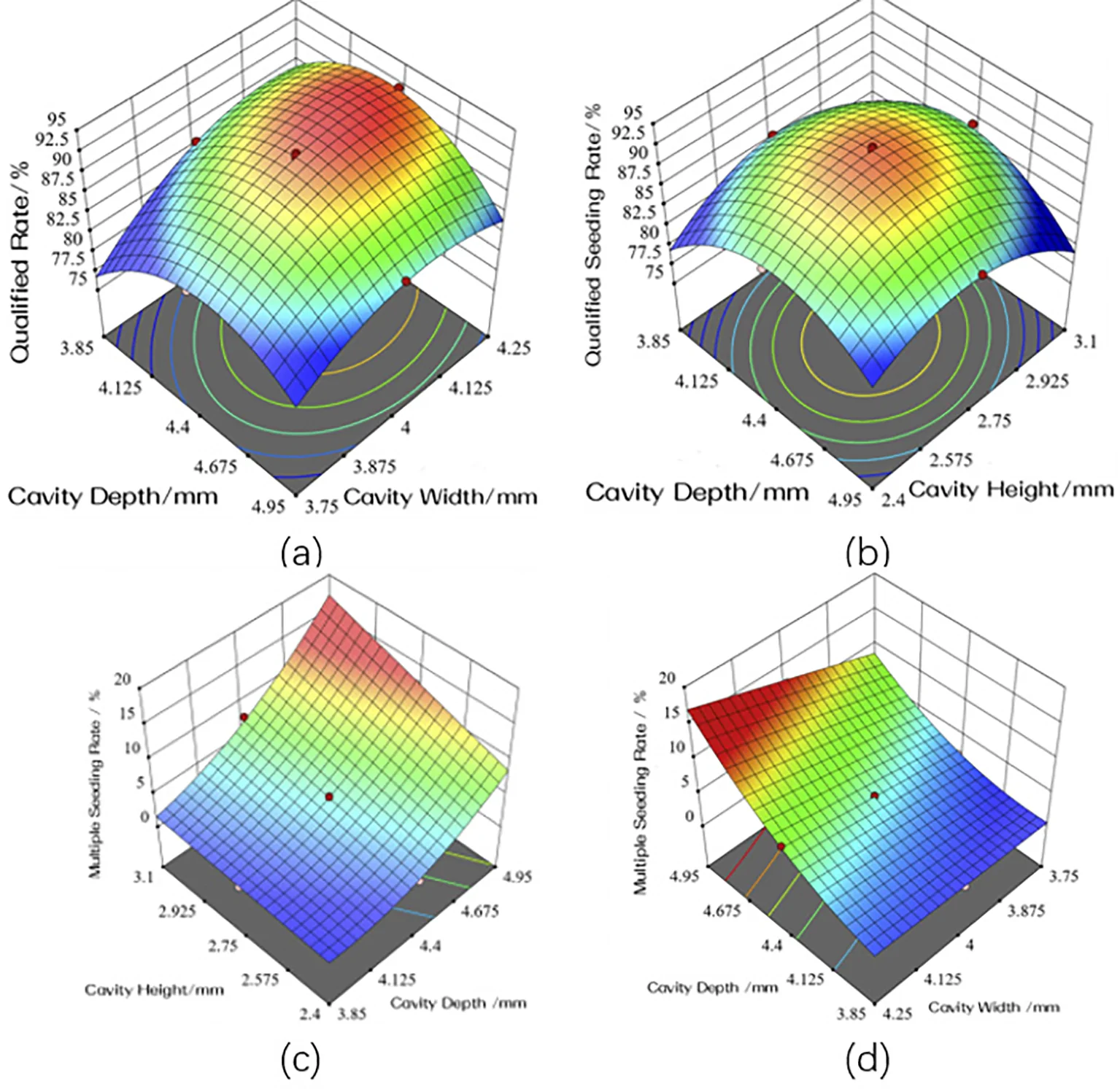
Effects of various factors on the qualification rate and re-seeding rate: **(a)** Effect of the interaction between effective width and effective depth on the qualification rate (C = 2.75 mm); **(b)** Effect of the interaction between effective height and effective depth on the qualification rate (B = 4.00 mm); **(c)** Effect of the interaction between effective height and effective depth on the re-seeding rate (B = 4.00 mm); **(d)** Effect of the interaction between effective depth and effective width on the re-seeding rate (C = 2.75 mm).

Multi-objective optimization is performed using Design-Expert 13 software. The values of effective cavity depth, width, and height are confined within the experimental ranges, and a nonlinear optimization function is established to maximize the qualified rate of seeds per hill while minimizing both the re-seeding rate and the miss-seeding rate. In practical foxtail millet sowing, re-seeding can be remedied by subsequent thinning, whereas miss-seeding leads to seedling gaps and causes irreversible losses in the field. Therefore, in the optimization module, maximizing the qualified rate is set as one objective; meanwhile, the highest priority is assigned to minimizing the miss-seeding rate, while a moderate weight is applied to reducing the re-seeding rate. The optimal parameter combination calculated by the software is as follows: effective cavity depth of 4.375 mm, effective cavity width of 4.131 mm, and effective cavity height of 2.736 mm. Under this set of structural parameters, the response surface model predicts a qualified rate of 92.54%, a re-seeding rate of 5.30%, and a miss-seeding rate of 2.16%. Considering machining accuracy and manufacturability, these optimized values are rounded to one decimal place. The final dimensions of the spoon cavity are determined as follows: effective depth of 4.4 mm, effective width of 4.1 mm, and effective height of 2.7 mm.

The optimized parameter combination was verified by three repeated DEM simulations in EDEM 2023 under the same settings and statistical methods as previously used, to validate the accuracy of the response surface model. The average qualified rate of seeds per hill, average re-seeding rate, and average miss-seeding rate obtained from the verification simulations were 91.94%, 4.44%, and 3.61%, respectively. To further interpret the simulation results, post-processing analysis of the particle motion characteristics and contact force distribution in the filling zone was conducted. The particle velocity vectors extracted from EDEM show that the downward-protruding rectangular deflector on each seed spoon generates a lateral push on the seed population as it sweeps through the seed mass, directing seeds toward the opposite side rather than simply accumulating in front of the spoon. This lateral flow prevents single-side dead-zone accumulation and ensures that the staggered spoons on both sides encounter a uniformly replenished seed population, which is essential for stable bilateral filling at low rotational speed. The contact force chain analysis indicates that within the spoon cavity, the 5–8 retained seeds form a relatively compact cluster at the cavity bottom, with contact forces concentrated in this bottom region; this force concentration stabilizes the seed cluster during the lifting phase. In contrast, excess seeds resting on the upper cleaning slope exhibit weak and discontinuous force chains, indicating that they are not firmly constrained and can readily slide back into the chamber under gravity as the spoon rotates upward. These observations are consistent with the working principle described in Section 3.2 and provide mechanistic support for the optimized cavity dimensions.

## 5 Bench scale experiments and field tests

### 5.1 Bench scale experiments

Bench-scale experiments were conducted in April 2025 on a self-developed seed metering performance test platform in the Engineering Laboratory of Intelligent Agricultural Machinery Equipment, Harbin Cambridge College. The foxtail millet cultivar Jinmiao K10 was used as the experimental material. To ensure consistency between the physical tests and simulation results, key components of the seed metering device were precisely manufactured via 3D printing using PLA material, and the cavity dimensions were configured according to the optimized structural parameters obtained previously.

The bench scale test setup is shown in **Figure 7**. The test bench mainly consists of a self made sand laying device, conveyor belt, stepper motors, seeding controller, seed metering device and an OpenMV4 H7 Plus camera. The horizontal movement of the conveyor belt was used to simulate the forward motion of a seeder. The sand laying device uniformly spread quartz sand over the conveyor belt surface to replicate actual field soil conditions and prevent foxtail millet seeds from bouncing and scattering after dropping. The seed metering device was driven by stepper motors, and the rotational speed of the seed metering disc was precisely adjusted by the seeding controller. To accurately evaluate the working performance of the seed metering device, periodic image acquisition was carried out by the OpenMV4 H7 Plus camera. When the seeded seeds were delivered to the monitoring area by the conveyor belt, the system captured and stored images in real time. After experiments were completed, the collected images were imported into a computer. The OpenCV library was invoked under a Python environment for subsequent image processing. The seed number per hill was identified and extracted, and then parameters such as centroid coordinates of seed groups and adjacent hill spacing were calculated. To validate the accuracy of the machine-vision seed counting, a random sample of 30 hill images from the bench test was independently counted manually by two experienced operators and compared with the algorithm output. The algorithm correctly identified the seed count in 28 out of 30 images (recognition accuracy=93.33%). The two misidentifications were caused by seed overlap within a single hill, where two touching millet seeds were incorrectly segmented as one. Given that the qualification threshold spans 5–8 seeds per hill (a range of 4 seeds), the ±1-seed recognition error is considered acceptable and does not materially affect the reported qualification, re-seeding, or miss-seeding rates. In addition to stepper-motor vibration, two further factors contribute to the discrepancy between DEM simulation and bench test results. First, natural variation in seed tri-axial dimensions within a single cultivar introduces stochastic filling behavior that the uniform DEM particle model cannot fully reproduce. Second, the FDM-printed PLA cavity dimensions carry a typical tolerance of ±0.1 mm, approximately 2–3% of the optimized 4.1 mm cavity width, which slightly alters the effective seed-holding volume. Together with stepper-motor micro-vibration, these factors account for the 1.27 percentage-point qualification-rate difference between simulation (91.94%) and bench test (90.67%) at 12 r/min.

**Figure 7:**
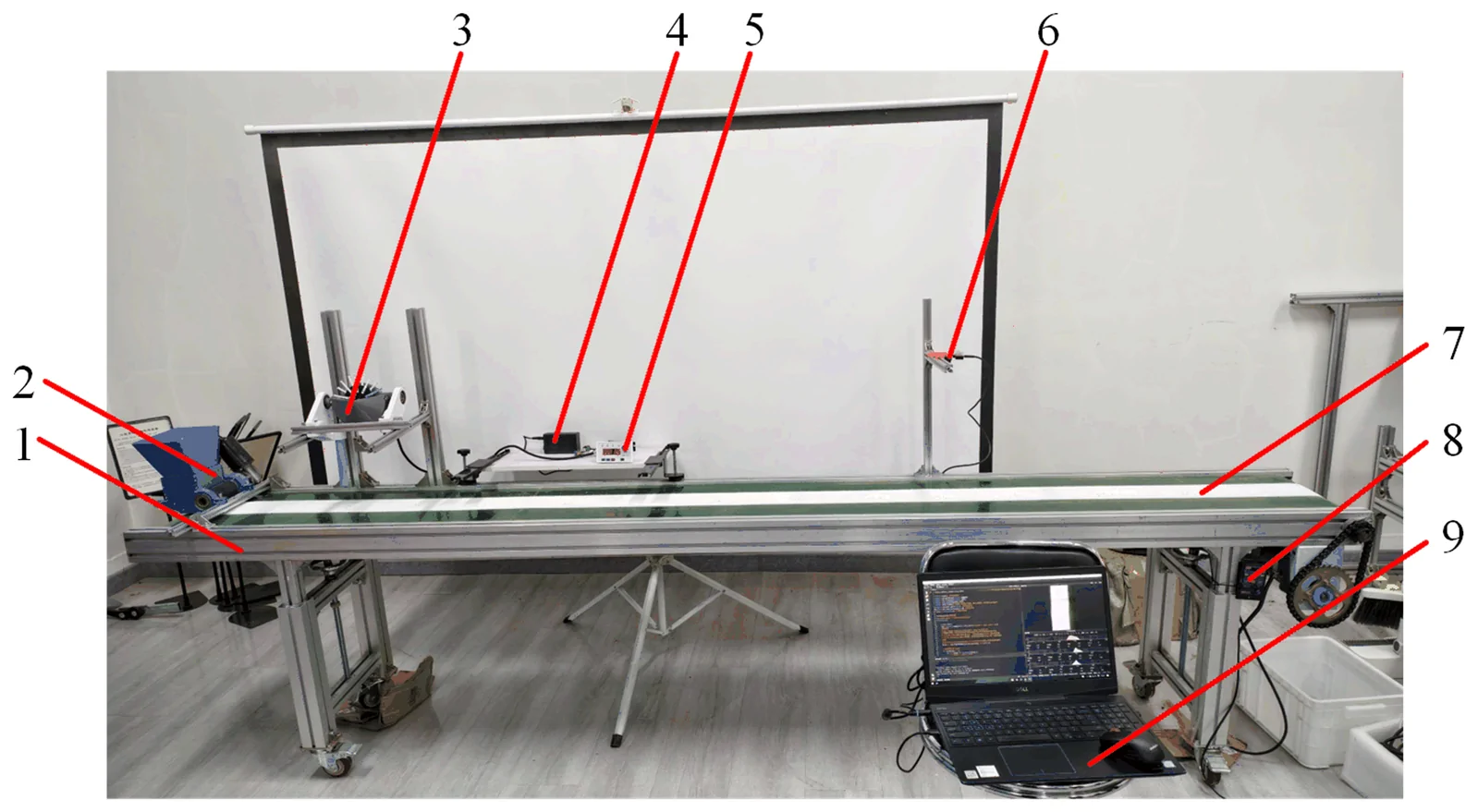
Bench test of the seed-metering device: (1. Conveyor belt 2. Sand spreading device 3. Seed-metering device 4. Power supply for the seed-metering device 5. Seeding controller 6. OpenMV4 H7 Plus camera 7. Quartz sand 8. Conveyor belt controller 9. Laptop computer).

Since the hand-pushed precision seeder for foxtail millet plot breeding designed in this study adopts electric drive and electronic control, the rotational speed of the seed metering disc must match the forward speed of the seeder during field operation. Based on the agronomic requirements for foxtail millet plot breeding, the theoretical hill spacing was set to 20 cm. The normal forward speed range of the hand-pushed seeder is generally from 0.4 m/s to 0.8 m/s. Using (Equation 1), the corresponding rotational speeds of the seed metering disc are calculated to range from 6 r/min to 12 r/min for operating speeds between 0.4 m/s and 0.8 m/s. For the rotational speed gradient tests, three speed levels (6 r/min, 9 r/min, and 12 r/min) were selected.

The performance evaluation indices for the bench-scale tests were consistent with those used in the previous simulation experiments. The number of seeds per hill was counted in the same manner. The seed dropping performance of 150 seed spoons was continuously recorded. The numbers of qualified hills (5–8 seeds per hill), re-seeding hills (more than 8 seeds per hill), and miss-seeding hills (fewer than 5 seeds per hill) were counted. The qualified rate of seeds per hill, re-seeding rate, and miss-seeding rate were then calculated from the measured data. The experimental results are listed in **Table 6**.

**Table 6:** Bench test results of the working performance of the seed-metering device.

Rotational speed/(r/min)	Qualification rate/%	Re-seeding rate/%	Miss-seeding rate/%
6	91.33	6.67	2.00
9	92.67	3.33	4.00
12	90.67	4.00	5.33

When the rotational speed of the seed metering disc is 6 r/min, seeds are stirred by the seed spoons for a longer duration inside the seed chamber, which ensures adequate seed filling. Excess seeds remain trapped inside the cavity and are not discharged in time, leading to a relatively high re-seeding rate. The qualified rate of seeds per hill peaks at 9 r/min. As the rotational speed further increases to 12 r/min, the linear velocity of the seed metering disc rises and shortens the effective filling time for seed entry. Meanwhile, seeds already carried in the cavity tend to slip off prematurely under gravity, centrifugal force, and micro vibration from the stepper motor. Consequently, the miss-seeding rate increases and the qualified rate of seeds per hill declines accordingly.

During the experiments, it was observed that the seed metering device on this test bench is driven by a stepper motor. The inherent stepping characteristics of the stepper motor cause vibration during disc rotation. Such physical micro vibration assists seed cleaning and causes redundant seeds that are loosely held at the cavity rim to fall off more readily. Meanwhile, seeds stirred up by the seed spoons settle to the bottom of the seed chamber under vibration. Therefore, this mechanical vibration effectively suppresses re-seeding during actual operation, but increases the miss-seeding rate to some extent.

At the rotational speed of 12 r/min, which matches the DEM simulation condition, the bench scale tests yielded a qualified rate of seeds per hill of 90.67%, a re-seeding rate of 4.00%, and a miss-seeding rate of 5.33%. Comparing these bench test measurements with the optimal simulation predictions obtained from the previous CCD optimization (91.94% qualified rate, 4.44% re-seeding rate, 3.61% miss-seeding rate), it can be concluded that the measured qualification rate is generally consistent with the predicted value, while the miss-seeding rate is slightly higher in the bench tests. This difference can be attributed to the EDEM 2023 discrete element model, in which the seed metering disc is set to perform smooth and uniform circular motion. However, in the bench tests, the pulsed vibration generated by the stepper motor improves the seed cleaning performance, causing grains in a critically balanced state to fall off more readily. The above experiments sufficiently verify the accuracy and reliability of the discrete element simulation model and the optimized parameters obtained from the response surface method.

According to the bench test data, the seed metering device achieves its optimal working performance at a rotational speed of 9 r/min. Within the manual operating speed range of 0.4–0.8 m/s for the hand pushed seeder, the qualified rate of seeds per hill remains above 90%, the re-seeding rate is no more than 7%, and the miss-seeding rate is lower than 6%. All these indices exceed the standard requirements specified in DB1307/T 397–2023 Technical Regulation for Precision Hill Seeding of Zhangza Millet, which requires that the qualified rate of seeds per hill for uncovered bottom hill seeding should be no less than 87%. These results demonstrate that the newly optimized seed metering device possesses good operational stability under practical operating conditions.

### 5.2 Field scale experiments

Field experiments were conducted on June 10, 2025, in the experimental field of the Engineering Laboratory of Intelligent Agricultural Machinery Equipment, Harbin Cambridge College, Harbin, Heilongjiang Province. The test plot was 12 m long and 1 m wide, with a soil moisture content of 13.31%. To verify the adaptability of the seeder for different foxtail millet cultivars, three varieties—Jinmiao K10, Zhangzagu-10, and Aogu-6—were selected based on the previously measured tri-axial dimensions of millet seeds. Prior to sowing, rotary tillage was carried out to adequately prepare the field soil to meet the sowing requirements of foxtail millet.

Field sowing tests were conducted to evaluate the practical working performance of the assembled spoon-wheel precision seeder for foxtail millet. During the tests, the seeder was manually pushed forward. The target hill spacing of 0.2 m was set via the seeding controller, and the forward speed was maintained at approximately 0.8 m/s. This upper working speed was deliberately selected for field validation because it represents the most demanding condition, where centrifugal interference and seed-shedding risk are highest; the bench tests had already confirmed qualification rates above 90% across the full 0.4–0.8 m/s range (**Table 6**). The controller automatically matched the rotational speed of the seed metering disc to 12 r/min accordingly. To avoid measurement errors caused by acceleration and deceleration during manual pushing, a 10 m steady traveling middle section was selected as the effective test length for each trial. Consistent with the previous bench tests, the qualified rate of seeds per hill, re-seeding rate, and miss-seeding rate were adopted as the evaluation indicators. During forward operation, an encoder collected real-time velocity signals from the ground wheel and fed the data back to the seeding controller. The controller then sent pulse, direction, and enable signals to the stepper motor driver. After receiving these control signals, the driver converted them and drove the stepper motor to rotate. In this way, the rotational speed of the seed metering disc precisely matched the forward speed of the seeder. The seed unloading efficiency of the seed metering device was evaluated for frequent seed re-placement scenarios in plot breeding. During the test, the unloading procedure for each trial was arranged as follows. First, the seeding controller cut off the pulse signals to the stepper motor, stopping disc rotation. Then, the entire seed metering device was manually inverted and shaken back and forth, so that residual seeds in the seed chamber fell into a collection bag by gravity. The total time from motor shutdown to complete seed evacuation and readiness for sowing the next cultivar was recorded. Each cultivar was tested three times, and the average value was adopted. In each trial, 100 consecutive hills within the 10 m steady traveling section were counted for the qualification, re-seeding, and miss-seeding rate calculations. The field sowing tests are shown in **Figure 8**.

**Figure 8:**
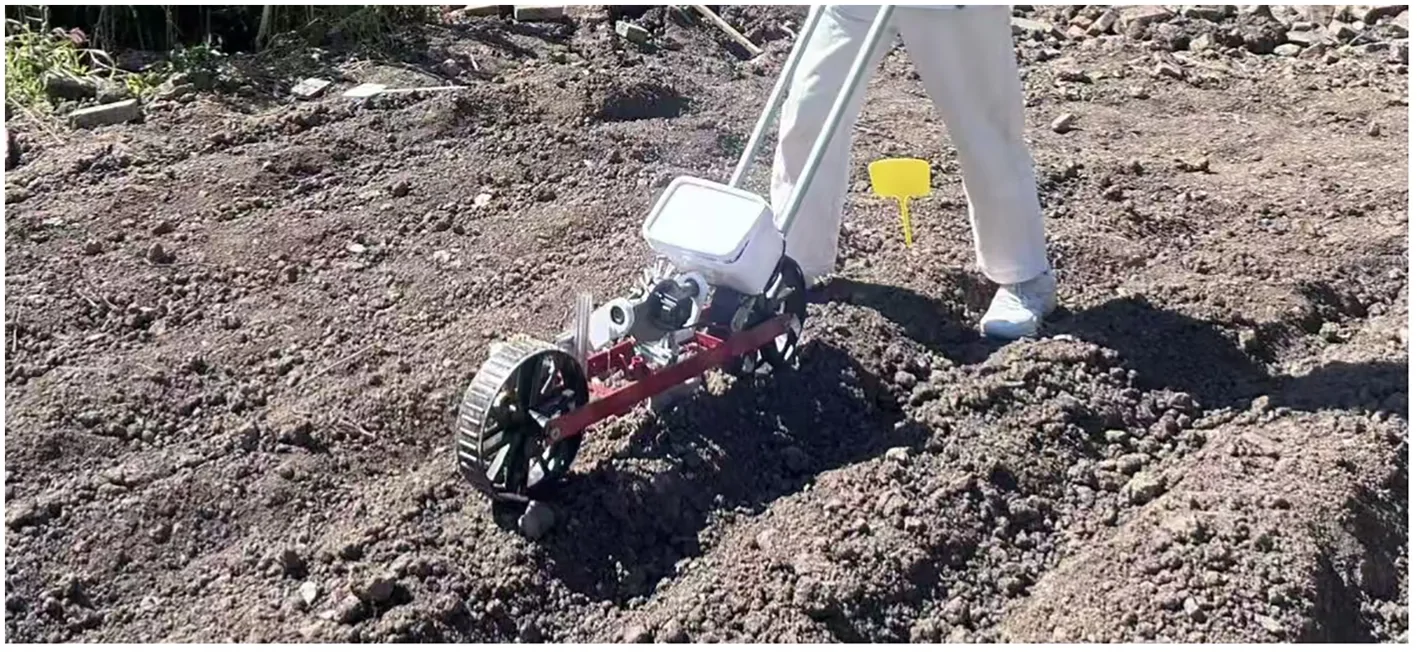
Field seeding test.

Statistical calculations for the field test results of Jinmiao K10, Zhangzagu-10, and Aogu-6 were performed separately according to (Equation 14) for the evaluation indicators. The statistical results are listed in **Table 7**.

**Table 7:** Field test results.

Variety	Qualification rate/%	Re-seeding rate/%	Miss-seeding rate/%
Jinmiao-K10	88	6	6
Zhangzagu-10	88	4	8
Aogu-6	90	6	4

**Table 7** shows that the developed spoon-wheel precision seeder for foxtail millet plot breeding achieves satisfactory performance for all three tested cultivars. All performance indicators consistently exceed the requirement of no less than 87% for the qualified rate of seeds per hill for uncovered-bottom hill seeding, as specified in DB1307/T 397–2023. Performance differences among cultivars are mainly attributed to the inherent physical properties of millet seeds. For Zhangzagu-10, the larger grain size and higher sphericity result in less adequate seed filling. Consequently, its re-seeding rate is slightly lower, while its miss-seeding rate is the highest among the three varieties. Aogu-6 exhibits the highest qualified rate of seeds per hill, owing to its plump grains and favorable dimensional uniformity. Jinmiao K10, used as the reference cultivar throughout this study, demonstrates moderate and stable performance across all evaluation indicators.

The average seed unloading time of the developed seeder was measured as 8.5 s, meeting the anti-seed-mixing requirements for plot breeding operations. Inspection after seed unloading showed that only negligible residual seeds remained (0–1 seed per test, mean = 0.67 seeds). Therefore, the seeder satisfies the frequent seed replacement and anti-seed-mixing demands of plot breeding practice.

## 6 Discussion

### 6.1 Effect of stepper-motor vibration on metering performance

The bench test revealed that the stepper-motor drive introduces micro-vibration during disc rotation. This vibration has a dual effect: it promotes the shedding of excess seeds loosely held at the cavity rim (thereby suppressing the re-seeding rate) but also causes critically balanced seeds within the cavity to dislodge prematurely (thereby increasing the miss-seeding rate). At 9 r/min, these two effects reach an optimum balance, yielding the highest qualification rate of 92.67%. At 6 r/min, the longer seed residence time in the chamber outweighs the vibration-assisted cleaning effect, resulting in a higher re-seeding rate of 6.67%. At 12 r/min, the shortened filling time combined with vibration-induced premature shedding increases the miss-seeding rate to 5.33%. This observation explains the discrepancy between the smooth-motion DEM model and the pulsed-motion bench system: the simulation assumes perfectly uniform rotation, while the physical device experiences stepwise micro-vibration that cannot be fully captured by the Hertz–Mindlin contact model without explicitly modeling motor dynamics.

### 6.2 Effect of seed morphology on filling performance

The three tested cultivars exhibited different metering performances that correlate with their seed geometric characteristics. Zhangzagu-10 produced the highest miss-seeding rate (8%) and lowest re-seeding rate (4%), which is attributed to its larger grain size and higher sphericity: spherical grains tend to roll rather than settle stably in the flat-bottomed cavity, reducing reliable single-layer retention. Aogu-6 achieved the highest qualification rate (90%) owing to its plump, dimensionally uniform grains that pack predictably in the optimized 4.1 mm-wide cavity. Jinmiao K10, whose mean tri-axial dimensions were used as the design basis for the cavity, showed moderate and stable performance (88% qualification). These results indicate that the optimized cavity dimensions are most suitable for seeds with width in the range of approximately 1.7–2.3 mm; cultivars significantly outside this range may require cavity width adjustment. To establish a quantitative design rule, the qualification rates of the three cultivars were correlated with their mean seed widths. A negative linear relationship was observed between qualification rate and seed width (R² ≈ 0.78), indicating that the optimized 4.1 mm cavity width is best matched to seeds with mean width ≤ 1.8 mm. This yields a cavity-to-seed width ratio of approximately 2.3:1, which provides sufficient clearance for reliable filling while preventing excess seeds from entering in a second layer. Although three cultivars are insufficient to claim universal adaptability across all foxtail millet germplasm, this ratio provides a preliminary design guideline for cavity dimensioning. Broader validation across a larger germplasm panel with more diverse seed sizes is identified as future work. Specifically, the three cultivars tested in this study represent only a small fraction of the >30,000 foxtail millet germplasm accessions conserved in the Chinese national genebank. Seed sizes in foxtail millet can range from approximately 1.5 mm to 2.5 mm in width, and the present validation covers only the 1.7–1.9 mm range. To rigorously establish broad adaptability, future work should test at least 10–15 cultivars spanning the full seed-size distribution, including glutinous and non-glutinous types, and quantify the qualification rate as a function of seed width to determine the effective operating range of the current cavity design. For germplasm with seed widths significantly outside the 1.7–1.9 mm range, cavity width adjustment following the 2.3:1 design rule identified in this study would be required.

It is also worth noting that the RSM model predicted a miss-seeding rate of 2.16% at the optimal parameters, whereas the three verification simulations yielded an average of 3.61%. This discrepancy arises from two sources. First, DEM seed filling is inherently stochastic: each simulation run generates a different random seed-packing configuration in the chamber, and only three verification runs were performed, so the sample size is limited. Second, the RSM quadratic regression model represents a smooth response surface that approximates a discrete physical process; the local minimum in the prediction may not exactly match the actual stochastic behavior of the system. Despite this difference, the qualification rate shows close agreement (92.54% predicted vs. 91.94% verified), and the overall trend of increasing miss-seeding rate with higher rotational speed is consistent between simulation and bench tests.

### 6.3 Speed-dependent metering mechanism

The rotational speed governs three competing physical processes: (1) seed filling time — lower speed allows longer residence in the filling zone, improving seed pick-up but also allowing more excess seeds to enter the cavity; (2) centrifugal force — higher speed increases the outward normal force component, reducing the normal support on the slideway and thus weakening sliding friction, which could theoretically accelerate dropping but also causes premature seed loss; and (3) vibration-induced shedding — as observed on the bench, higher step frequency intensifies micro-vibration. The combined effect produces the observed hump-shaped qualification-rate curve peaking at 9 r/min. Within the manual pushing speed range of 0.4–0.8 m/s (6–12 r/min), the qualification rate remains above 90%, confirming that the device is robust to the natural speed variation of hand-pushed operation.

### 6.4 Residue-free cleaning performance and breeding implications

The average seed unloading time of the developed seeder was measured as 8.5 s (mean of three repeated tests, standard deviation = 0.6 s), meeting the anti-seed-mixing requirements for plot breeding operations. The standardized unloading procedure was as follows: the controller cut off pulse signals to stop disc rotation; the entire seed metering device was manually inverted to 180° and shaken at approximately 1.5 Hz through a 45° angular amplitude until all visible seeds exited the chamber; and the total time from motor shutdown to readiness for the next cultivar was recorded. After gravity unloading, the seed chamber was brushed once with a soft nylon brush, and all residual seeds were collected, counted, and weighed. The residual seed counts across three tests were 0, 1, and 1 seed (mean = 0.67 seeds; residual mass < 0.002 g), confirming effective residue-free cleaning. Therefore, the seeder satisfies the frequent seed replacement and anti-seed-mixing demands of plot breeding practice. The performance improvements delivered by this device translate directly into higher quality breeding experiments. In plot breeding, each hill represents a single experimental unit, and a miss-seeding event creates a missing data point that cannot be recovered post-emergence. Reducing the miss-seeding rate from the commonly reported 8–10% for conventional millet seeders to 4.00% in this work decreases the proportion of missing experimental units by approximately 50%, thereby reducing the standard error of cultivar yield comparisons and improving the statistical power of breeding trials. Furthermore, cross-contamination during cultivar changeover is a critical source of experimental error in plot breeding: even a single residual seed from the previous cultivar can grow into a misclassified plant that biases phenotypic evaluation. The residue-free cleaning mechanism (mean residual = 0.67 seeds per changeover, <0.002 g) effectively eliminates this error source, ensuring that each plot contains only the intended cultivar and that phenotypic measurements are not confounded by off-type plants. In terms of operational efficiency, the 8.5 s residue-free clearing time reduces the cultivar changeover duration by an order of magnitude compared with conventional disassembly-and-brush cleaning, which typically requires 2–5 min per plot. For a breeding nursery containing 200–500 entries per day, this translates to a time saving of 4–15 h per sowing season, allowing larger germplasm panels to be evaluated within the narrow optimal sowing window. The consistent hill-drop metering (5–8 seeds per hill, qualification rate ≥88% across three cultivars) also improves data reliability by ensuring uniform plant density across plots, which is a prerequisite for fair comparison of yield and agronomic traits among cultivars.

The PLA 3D-printed components used in this study are functional prototypes validated for a single growing season (approximately 50–80 h of cumulative operation). Several factors limit the long-term field durability of PLA. First, abrasive wear: foxtail millet seeds have a hard seed coat with a silica-rich outer layer, and repeated contact during filling, cleaning, and dropping gradually abrades the cavity surfaces. Based on typical PLA wear rates under seed-contact conditions, the effective cavity width may increase by 0.1–0.2 mm after 100 h of operation, which could shift the qualification rate by approximately 2–3 percentage points. Second, environmental degradation: PLA is susceptible to UV-induced embrittlement and hydrolysis under prolonged exposure to sunlight and high humidity, which can cause surface cracking after 2–3 growing seasons. Third, the coefficient of restitution and SFC between PLA and millet seeds may change as the surface roughness evolves during wear, potentially altering filling and dropping behavior. For long-term commercial deployment, seed-contact surfaces (seed spoons and chamber inner wall) should be replaced with more abrasion-resistant materials such as Nylon 12 (PA12) via selective laser sintering, or sintered stainless steel for high-wear components. A periodic inspection schedule is recommended: cavity dimensions should be measured every 50 h of operation, and components replaced when dimensional deviation exceeds ±0.15 mm. Two further practical limitations should be noted for field deployment. First, under high-humidity soil conditions (soil moisture >20%), moist soil particles may adhere to the seed-dropping window and partially obstruct seed exit. In the present field tests (soil moisture = 13.31%), no blockage was observed; however, for deployment in regions with higher soil moisture or during early-spring sowing, a removable scraper or air-purge nozzle at the dropping window would be a worthwhile design addition. Second, field operation introduces ground-induced vibration through the hand-pushed frame, which differs from the controlled bench environment. While the bench tests already incorporate stepper-motor vibration, additional field vibration from uneven ground may slightly increase the miss-seeding rate. Future work should evaluate device performance under field vibration conditions using a tractor-mounted test rig with accelerometer monitoring.

### 6.5 Comparison with existing seed-metering devices

**Table 8** compares the present device with four recently reported small-grain seed-metering devices. Zheng et al. (2024) reported a spoon-wheel spinach device with improved filling uniformity, but its brush cleaning mechanism causes seed damage and is unsuitable for plot breeding where cross-contamination must be eliminated. Kou et al. (2024) achieved improved millet filling through air assistance, but the internal airflow channels retain residual seeds and prevent rapid cleaning between cultivars. Zhang et al. (2024) developed an electromagnetic vibration millet seeder with precise small-amount metering, but it operates as a continuous drill and cannot deliver the discrete hills required for plot breeding evaluation. Zhang (2014) designed a low-rate foxtail millet seeder, but its qualification rate was below 85% and it lacked a dedicated cleaning mechanism. For plot-breeding-specific devices, Deng et al. (2024) developed a pneumatic–seed-spoon interactive precision maize seed-metering device for plot planting that achieved high metering accuracy, but its pneumatic system requires airtight components and a compressed-air supply, which adds complexity and cost for small-scale breeding nurseries. The comprehensive review by Shang et al. (2021) identified residue-free cleaning and cultivar adaptability as the two most critical unmet needs for plot-breeding seeders. The present device directly addresses both needs: the flip-type gravity unloading achieves residue-free cleaning in 8.5 s without disassembly, and the qualification rate remains ≥88% across three foxtail millet cultivars with differing seed sizes. Unlike pneumatic plot seeders, the purely mechanical design requires no compressed air or complex control electronics, making it suitable for deployment in remote breeding stations with limited infrastructure. It should be acknowledged that direct head-to-head bench testing against competing devices was not feasible in this study, as the reference devices were not available in our laboratory. The comparison in **Table 8** is therefore based on performance data reported in the original publications, which may have been obtained under different test conditions (e.g., different seed lots, rotational speeds, or evaluation protocols). To enable a fairer direct comparison, future work will conduct parallel bench tests of the present device alongside a commercially available plot seeder under identical seed material and operating conditions, quantifying differences in qualification rate, miss-seeding rate, seed damage rate, and cleaning time under controlled conditions. The present device achieves a 92.67% qualification rate, 4.00% miss-seeding rate, and 8.5 s residue-free clearing, while maintaining hill-drop metering specifically for foxtail millet plot breeding.

**Table 8:** Comparison of the present device with reported small-grain seed-metering devices.

Device	Crop	Re-seeding rate	Qualification rate/%	Residue-free cleaning time	Miss-seeding rate/%
Zheng et al. (2024)	Spinach	~85%	Not reported	Not reported	High (brush)
Kou et al. (2024)	Foxtail millet	~88%	~6%	Not reported	Low
Zhang et al. (2024)	Foxtail millet	Not hill-drop	Not applicable	Not reported	Low
**Present work**	**Foxtail millet**	**92.67%**	**4.00%**	**8.5 s**	**None (gravity)**

Bold values indicate the results of the present work (the seed-metering device developed in this study).

## 7 Conclusions

1)To address the high miss-seeding rates in precision seeding of small grains and the seed mixing caused by seed replacement and machine cleaning in foxtail millet plot breeding tests, a bilateral staggered spoon-wheel precision seed metering device was proposed. By arranging seed spoons alternately on both sides of a horizontal shaft and adopting a side-opening seed dropping structure, the proposed device reduces the rotational speed of the seed metering disc while maintaining the seed dropping frequency. Accordingly, seed splashing induced by centrifugal force on tiny grains is effectively alleviated. Combined with a semi-closed seed chamber design, gravity-driven gentle seed delivery is realized during seeding operation, and residue-free seed unloading is achieved through overall overturning for seed replacement.

2)A discrete element simulation model of the seed metering device and foxtail millet seeds was established. Single-factor experiments and central composite design (CCD) response surface tests were conducted to determine the optimal structural parameters of the seed spoon cavity: effective cavity depth of 4.4 mm, effective cavity width of 4.1 mm, and effective cavity height of 2.7 mm. Simulation verification showed that under the optimal structural parameters, the qualified rate of seeds per hill, re-seeding rate, and miss-seeding rate were 91.94%, 4.44%, and 3.61%, respectively.

3)Bench and field tests were conducted to verify the seeding performance and cultivar adaptability of the seed metering device. The bench tests showed that the seeder achieved optimal performance at 9 r/min (forward speed of 0.6 m/s). Under this condition, the qualified rate of seeds per hill reached 92.67%, the re-seeding rate was 3.33%, and the miss-seeding rate was 4.00%. Field tests indicated that at an operating speed of 0.8 m/s, the qualified rates of seeds per hill for Jinmiao K10, Zhangzagu-10, and Aogu-6 were all higher than 88%. These values exceed the 87% requirement specified in DB1307/T 397–2023. The average seed unloading time was 8.5 s, with no residual seeds inside the seed chamber after seed replacement, which satisfies the agronomic requirements of high efficiency, high precision, and seed mixing prevention for plot breeding operations.

## Data Availability

The original contributions presented in the study are included in the article/supplementary material, further inquiries can be directed to the corresponding author/s.
